# Usage and effectiveness of strategies to sit less and move more: evaluation of the BeUpstanding™ national implementation trial

**DOI:** 10.1186/s12966-025-01761-4

**Published:** 2025-05-28

**Authors:** Genevieve N. Healy, Elisabeth A.H. Winkler, Samantha K. Mulcahy, Charlotte L. Brakenridge, Ana D. Goode

**Affiliations:** 1 Present Address: School of Human Movement and Nutrition Sciences, Health and Wellbeing Centre for Research Innovation, The University of Queensland, Brisbane, QLD 4072 Australia; 2 Present Address: Centre for Work, Organisation and Wellbeing, Griffith University, 4111 Nathan, QLD Australia

**Keywords:** Workplace health promotion, Office workers, Sedentary behaviour, Physical activity, Intervention

## Abstract

**Background:**

Addressing prolonged workplace sitting is an identified priority. A national implementation trial of BeUpstanding™ — an online workplace intervention supporting teams of desk-based workers to sit less and move more — successfully reduced worker sitting time in a large sample of workers. However, it is unclear which strategies workers used to sit less and move more, how usage changed following intervention, and how this related to changes in work activity and sitting.

**Methods:**

BeUpstanding collected data from staff before and after the 8-week champion-delivered intervention regarding self-reported work behaviours (percentage of worktime sitting and moving; percentage sitting in prolonged bouts) and the usage (0 = never to 4 = always/nearly always) of 21 sit less/move more strategies (13 emphasised ‘move more’). Strategy usage during versus before intervention were compared via linear mixed models. Strategy usage (0–4) and changes (-4–4) were tested in relation to post-intervention behaviours and behaviour changes using linear mixed models. Interaction tests and conditional inference trees compared strategies in their effectiveness.

**Results:**

Across 1614 staff (43.1 ± 11.3 years; 66% female) from 66 workplaces, the number of strategies used at least sometimes averaged 9.56 (SE = 0.19) pre-intervention. Strategy usage increased significantly (*p* < 0.05) in the number of strategies used (2.45 [95% CI: 2.18, 2.73], *p* < 0.001), mean strategy usage (0.37 [0.33, 0.41], *p* < 0.001), move-more strategy usage, and for every strategy except active travel. Every strategy was used by > 10% of staff following intervention. Strategy usage and changes were significantly associated with all behaviours and behaviour changes (all *p* < 0.01). There were significant differences in the strength of these associations between strategies (*p* < 0.05) and for move-more strategies versus other strategies (*p* < 0.05); however, no strategies were statistically counterproductive. Conditional inference trees identified various combinations of strategies whose usage predicted outcomes.

**Conclusions:**

All strategies showed acceptability (used by > 10%), most were modifiable (increased with intervention), and, to varying degrees, their usage was associated with work behaviours. Findings suggest all 21 strategies are suitable for Australian desk-based workers to select based on personal and contextual fit. Strategies most strongly linked with all behaviours or targeted behaviours (i.e., increasing movement) might be emphasised to enhance effectiveness.

**Trial registration:**

ACTRN12617000682347. The trial was prospectively registered on the 12th May, 2017 (ACTRN12617000682347) before the soft launch online and last updated on the 11th June 2019, before the national implementation trial recruitment commenced (12th June, 2019).

**Supplementary Information:**

The online version contains supplementary material available at 10.1186/s12966-025-01761-4.

## Background

Sedentary time (too much sitting) amongst desk-based workers is high, averaging 76% of their workday [1]. The corresponding risks for health and wellbeing associated with high sedentary time [2, 3] has led to this behaviour being identified as an emerging occupational health and safety risk [4]. Accumulating sedentary time in prolonged, unbroken bouts (e.g., of 30 min or more) may be particularly detrimental [5], with both public health (e.g [6, 7])., and occupational [8] guidelines recommending regularly breaking up sedentary time. Numerous studies have now reported on the effectiveness of interventions addressing workplace sedentary time [9–11], finding that they succeed but to varying degrees and with often relatively limited impact on moving time, despite the dual promotion of both sit less and move more in these interventions [12–14]. Underpinning this variable success may be the specific strategies that workers use to achieve these changes.

Strategy usage has been reported upon by very few studies [15–17], with all studies to date conducted in limited contexts (like a single organisation or sector). It is important to determine in a wide range of diverse workplaces the strategies that are: used by workers (showing they are acceptable); used to a greater degree with intervention (showing they are modifiable); and, linked with behaviour (showing they are effective). In particular, evaluating strategies that specifically target moving may be particularly relevant to being able to improve upon the limited impact sit less/move more interventions have had on moving [16–18]. Stephens et al., reported on the sit less/move more strategies participants self-nominated to use as part of the Stand Up Victoria cluster-RCT, with 82 unique strategies described [16]. Strategy choice impacted on behaviour change, with nominating to use the sit-stand workstation for three or more hours a day and choosing task-based strategies to stand up (e.g., stand up at the end of a phone call) emerging as the most important [16]. This strategy list informed development of a questionnaire on strategy use which was then tested in an intervention trial [17]. Here, significantly greater reductions in prolonged sitting time (sitting for 30 min or more at a time) were seen with increased use of stairs, standing meetings, and using a central bin, while group physical activity sessions and participating in a workplace pedometer/walking competition were significantly associated with increased stepping [17]. In both studies, an increase in strategy use resulted in desired behaviour changes [16, 17], suggesting that a range of strategies may be effective. However, both studies were conducted within a single organisation with workers in relatively similar job roles, and strategy usage is expected to be highly contextual (e.g., a strategy of taking the stairs requires the availability of stairs). To provide broadly applicable guidance on acceptable, modifiable, and effective strategies, it is important to examine a diverse range of workplaces and workers, ideally where these workplaces can self-select the strategies they choose to promote.

The present study provides exactly such evidence, using data from the national implementation trial of BeUpstanding. BeUpstanding is an evidence-based, champion-led, online workplace health promotion initiative that aims at supporting teams of desk-based workers to sit less and move more through raising awareness and creating a supportive culture for change [18]. Findings from the national implementation trial of BeUpstanding shows the intervention achieves this aim, with significant improvements in workplace activity levels, including reductions in prolonged sitting and increases in movement, reported [19]. Part of the BeUpstanding evaluation includes assessment of self-reported staff sit less/move more strategy use and workplace behaviour (sitting, sitting accumulation, moving) before and at the end of the 8-week intervention. Using this data, which has been collected from a wide range of industries and sectors [20], the aims of this study were to: (1) describe the usage of sit less/move more strategies both prior to and during intervention; (2) determine whether the BeUpstanding intervention was effective in promoting an increase in strategy usage; (3) quantify how strategy usage relates to behaviours and behavioural changes; and, (4) examine potentially effective combinations of strategies. In addressing these aims, the findings are expected to help inform workplace sit less/move more interventions to choose a ‘menu’ of strategies to offer by identifying strategies that should be: excluded (i.e., counterproductive, usage is non-modifiable, or there is no usage [possibly not acceptable]); emphasised (i.e., highly effective; effective and able to be used by many people; or, effective and shows large increases in usage); and, optionally included, depending on how large of a menu is able to be offered (i.e., everything else that can be modified, has some usage, and has some effectiveness).

## Methods

### Study design and participants

The present study is a secondary analyses of data collected as part of the national implementation trial of the BeUpstanding intervention. This trial, which had a recruitment period between June 19th 2019 and September 30th 2021, used a hybrid implementation-effectiveness design and repeated cross-sectional evaluations of staff before and after the intervention to establish effectiveness [18]. Ethical approval was from The University of Queensland Human Research Ethics Committee (approval #2016001743) with all participants providing informed consent.

### BeUpstanding intervention

The BeUpstanding intervention is delivered by a workplace champion (identified by the workplace), ideally with strong support from leadership. The evidence-base underpinning BeUpstanding [18], the multi-phase research-to-practice translation process [21–23], details of the intervention [19], the implementation trial methods [18] and the primary outcomes [19] have been reported in detail, along with the TREND and TIDieR checklists [19]. In brief, the intervention involves seven core components. The most critical component is the staff information and consultation workshop (or equivalent) where staff are provided education on the benefits of sitting less/moving more and then encouraged to collectively decide on the three (or more) strategies they want to do as a team to sit less/move more. These team-level strategies are then promoted by the champion over 8-weeks using resources provided within the online toolkit. This participative approach, which meant each intervention was unique for each team, intends to ensure that staff have ownership over the changes and are collectively contributing to changing the culture and awareness around sitting less and moving more in the workplace. While the BeUpstanding resources encourage teams to choose strategies across the hierarchy of control [24], there is no prescribed strategy list and team-level strategies chosen may or may not be similar to the list of strategies staff are asked to self-report their usage on. Intervention messaging encourages workers to aim for 50% or less of their workday sitting, with the remaining time spent standing or moving.

The national implementation trial of BeUpstanding targeted recruitment of champions willing to deliver the BeUpstanding intervention to teams in Australia comprised predominantly of desk-based workers with high levels of sitting. Access to sit-stand workstations was not an eligibility criteria. Ultimately, 111 champions for 94 participating teams from 82 workplaces that were part of 65 organisations were eligible and participated in the trial [19]. The trial recruited a diverse range of workplaces across every state and territory in Australia, covering (to varying degrees) every key priority sector identified by the partners (small business, public sector, remote, call centre, blue collar). Participating organisations represented 13 of the 19 standard Australian industries [25], primarily from Health Care and Social Assistance (e.g., Hospitals) and Professional / Technical and Scientific Services (e.g., engineers). This secondary analysis mainly focuses on the 69 teams from 66 workplaces in 58 organisations that participated in the pre- and post-intervention staff evaluations, providing data regarding the strategies staff used and staff behaviours, and their main champions (*n* = 69).

### Data collection and measures

BeUpstanding is hosted on a bespoke learning, research and management system (Wildfyre™). Workplace champions use the toolkit hosted on the system to access the resources required to deliver and evaluate the intervention. As part of the intervention, champions are required to complete a profile survey to unlock the toolkit, with this survey capturing organisational and team-level data. Using the toolkit, champions are then asked to send out pre-intervention and post-intervention surveys for staff to complete, with these surveys capturing staff behaviour and staff strategy usage information. The toolkit also contains a workplace audit for champions to complete as part of the needs analysis. Champions were contacted by the research team as part of the implementation trial, both to provide implementation support and to also collect implementation data.

### Staff strategy usage

Staff reported how often (0 Never or Not applicable / 1 Rarely / 2 Sometimes / 3 Often / 4 Very often or always) they used each of a standard list of 21 strategies to sit less and move more at work in the last month. Of these, 13 strategies, termed ‘move-more strategies’, had a definite move-more component (in addition to sitting less), while the remaining strategies were likely to either result in only increased standing, or could result in standing, moving, or both depending how they were enacted (such as the ‘wear comfortable footwear’ strategy). This strategy list had been iteratively developed from previous trials [16, 17, 23, 26], with each strategy question detailed in full in Supplemental Table 1. In addition to strategy usage (0–4), a binary classification of the strategy being used was considered (strategy used at least sometimes, yes/no). Summary scores of number of strategies used at least sometimes, and mean strategy usage (0–4) were calculated across all 21 strategies, and for the subset of 13 move-more strategies of both of these. Internal consistency (Cronbach’s α) was acceptable (i.e., 0.7–0.8) for strategy usage (21 items), but was slightly lower (0.6–0.7) for move-more strategy usage (13 items) (Supplemental Table 1).

### Staff behaviours

Behavioural outcomes that were considered in relation to strategy usage were: sitting and moving (each as a % of the workday), and prolonged sitting accumulation (% of sitting at work accumulated in unbroken, continuous bouts of 30 min or more). These were measured in pre- and post-intervention staff surveys, using the Occupational Sitting and Physical Activity Questionnaire [27], and a single item regarding prolonged sitting accumulation [28].

### Other measures

Demographic data were collected in the pre- and post-intervention staff surveys, with the first available response used. Data pertaining to the organisation, workplace and team were drawn from the team champion’s responses to champion profile surveys, workplace audits, or questions asked in implementation check phone calls. Where data were unavailable, responses were used from another champion from the same workplace with relevant knowledge about the team, workplace, or organisation. Organisational attributes (main industry, sector [public / non-profit / private], and size) were checked against online records.

### Statistical analyses

Analyses were performed in STATA (StataCorp, LLC) and significance was set at *p* < 0.05 (two-tailed). Characteristics of participating workplaces, teams and staff were described as mean ± SD or n (%) for those in the trial overall and the subset able to be used in this analysis (because they provided data required for analysis). The odds of teams or staff of being included (yes/no) were compared based on workplace and team characteristics respectively as odds ratios (with 95% confidence intervals). Strategy usage before the BeUpstanding intervention (from pre-intervention surveys) and during BeUpstanding (from post-intervention surveys) as well as changes in usage were described for each strategy using descriptive statistics (Aim 1). Changes in strategy usage over the course of the intervention were tested using linear mixed models to account for repeated measures and clustering (Aim 2), with outcome variables being: the number of strategies (0–21) and move more strategies (0–13) used at least sometimes; mean strategy usage (0–4); and, mean move more strategy usage (0–4). Linear mixed models contained random intercepts for workplace cluster and staff (nested within cluster), as well as fixed effects for timepoint, and for the potential confounders as described in the main outcomes paper [19].

**Table 1:** Characteristics of participating workplaces, teams and staff

	All trial (*n* = 94 teams) ^a^		In analysis (*n* = 69 teams)	
	n	n (%) / M ± SD	n	n (%) / M ± SD
**Teams**				
State	94		69	
New South Wales		17 (18.1%)		13 (18.8%)
Australian Capital Territory		5 (5.3%)		4 (5.8%)
Victoria		14 (14.9%)		11 (15.9%)
Queensland		43 (45.7%)		30 (43.5%)
South Australia		7 (7.4%)		4 (5.8%)
Western Australia		6 (6.4%)		5 (7.2%)
Tasmania		1 (1.1%)		1 (1.4%)
Northern Territory		1 (1.1%)		1 (1.4%)
Organisation Size	94		69	
Small (< 20)		11 (11.7%)		10 (14.5%)
Medium (20–199)		13 (13.8%)		13 (18.8%)
Large (200–1999)		24 (25.5%)		16 (23.2%)
Very large (2000+)		46 (48.9%)		30 (43.5%)
Sector	94		69	
Public		48 (51.1%)		30 (43.5%)
Nonprofit		14 (14.9%)		13 (18.8%)
Private		32 (34.0%)		26 (37.7%)
Regional staff included (Yes)	94	32 (34.0%)	69	20 (29.0%)
Call-centre staff included (Yes)	94	6 (6.4%)	69	1 (1.5%)
**Staff** ^b^				
Age	2004	42.9 ± 11.4	1614	43.1 ± 11.3
Female	2131	1386 (65.0%)	1614	1068 (66.2%)
Job classification	2043		1614	
Employee		1399 (68.5%)		1082 (67.0%)
Middle management		167 (8.2%)		144 (8.9%)
Upper management		477 (23.3%)		388 (24.0%)
Full-time employment (Yes)	2043	1678 (82.1%)	1614	1308 (81.0%)
Work hours per week	2043	38.2 ± 9.0	1614	37.9 ± 8.9

^a^ Main champion for team only

^b^ SD corrected for clustering using linearized variance (STATA survey commands)

Associations of strategy usage (0–4) with work behaviours (Aim 3), were examined using linear mixed models. Strategy usage (0–4) was considered in relation to post-intervention behaviour and behaviour changes, and changes in strategy usage were considered in relation to change in behaviours. Rather than using summary scores, each of the 21 strategies were included, with random intercepts for strategy (1/2/…21) used to handle the repeated data and random intercepts for cluster used to accommodate the workplace clustering. Models also included fixed terms for strategy (1/2/…21), usage of the strategy (0–4), age, sex, small-medium enterprise (yes/no) and sector (public/nonprofit/private). Then, to test whether associations of strategy usage with outcomes differed by whether the strategy had a move more focus or not, or varied between the different strategies, interaction terms were added to the model. Marginal means from these interaction models were then used to report the associations separately for move more strategies and for each of the strategies.

Conditional inference trees were developed to identify a small set of strategies that may be most predictive of post-program behaviours and changes in behaviours (Aim 4). Unlike the previous models, which test associations, conditional inference trees add a different perspective on the findings by also allowing consideration of combinations of variables that may best predict the outcomes. Conditional inference trees, fit using the partykit R package [29], were chosen over traditional decision trees because they use inferential statistical testing (rather than heuristic measures such as information criteria, for example) to select variables, which limits excess growth and improves the generalisability of results. These models not only select the relevant variables, but also the thresholds at which differences are most apparent.

## Results

### Participant and team characteristics

BeUpstanding trial participants had an average age of 42.9 ± 11.4 years, were mostly female (65.0%), employees (68.5%) rather than management, and mostly worked full time (82.1%), for an average 38.2 ± 9.0 work hours per week (Table 1). The only staff characteristic that differed between trial participants who were included versus excluded from analyses in this paper (Supplemental Table 2) was age, resulting in only a slightly higher mean age seen in the included participants (43.1 ± 11.3 years) relative to the mean for trial participants in general. Teams participating in the trial (*n* = 94) were from all states and territories across Australia (Table 1) and mostly from very large (2000 + workers) organisations (48.9%). About half of participating teams were from the public sector (51.1%), just over a third included regional workers (34.0%), while very few included call-centre workers (6.4%). The odds of teams being included in this secondary analysis (yes/no) (Supplemental Table 2) varied significantly by organisational size (*p* = 0.018) and whether the team included call centre staff (*p* = 0.012). They also tended to vary by sector (*p* = 0.053) and whether the team included regional staff (*p* = 0.089), with the highest odds of inclusion respectively seen for teams: from small-medium enterprises; without call centre staff; from the non-profit sector; and without regional staff.

**Table 2:**
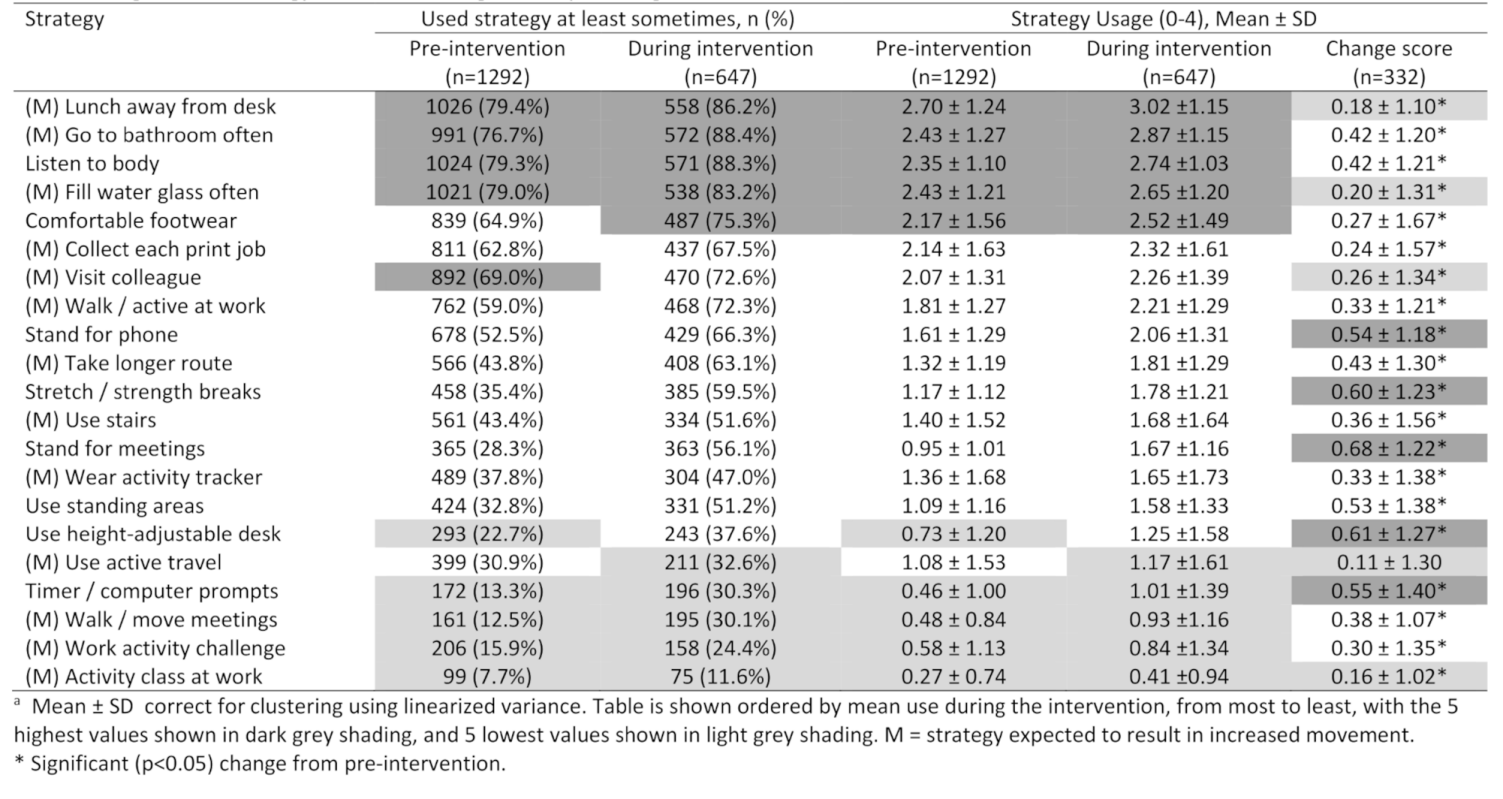
Usage of each strategy before and during the beupstanding intervention ^a^

### Aim 1: describing strategy usage before and after intervention

Usage of each strategy prior to BeUpstanding and during BeUpstanding are described in Table 2, along with changes in usage of each strategy. Prior to BeUpstanding, the strategy used at least sometimes by the most participants was taking lunch away from the desk (79.4%). Every strategy was used at least sometimes by 10% or more of participants during the intervention. The strategies with the highest usage (0–4) during the intervention were taking lunch away from the desk (3.02 ± 1.15); using a more distant bathroom (2.87 ± 1.15); listening to the body as a prompt to move (2.74 ± 1.03); using water as a reason to move (2.62 ± 1.20); and, wearing comfortable footwear (2.52 ± 1.49). These were all used least sometimes by over 75% of participants. The five strategies with the lowest strategy usage during the intervention were: activity classes (0.41 ± 0.94); activity challenges (0.84 ± 1.34); walking meetings (0.93 ± 1.16); timer / computer alerts (1.01 ± 1.39); and, active travel (1.17 ± 1.61). These strategies were used at least sometimes by less than one-third of participants.

### Aim 2: changes in strategy usage following the intervention

Changes in strategy usage following the BeUpstanding intervention are shown in Table 3. Adjusted results, which were nearly identical to unadjusted results, showed the number of strategies used at least sometimes increased significantly over the intervention by an average of 2.45 strategies out of 21 (95% CI: 2.18, 2.73), from a mean 9.56 (SE = 0.19) prior to the intervention. Mean strategy usage (0–4) increased significantly over the intervention by 0.37 points (95%CI: 0.33, 0.41), from a pre-intervention mean of 1.48 (SE = 0.03). Move more strategy usage showed similar results (Table 3). The data reported in Table 3 provide context to these average changes, showing they occurred alongside increased usage of every strategy, with all increases being statistically significant except for active travel. Notably, the five largest changes were seen for: standing for meetings (0.68 ± 1.22); using a height-adjustable desk (0.61 ± 1.27); stretching (0.60 ± 1.23); timer / computer alerts (0.55 ± 1.40); and, standing to use the phone (0.54 ± 1.18).

**Table 3:** Changes in strategy usage across the beupstanding intervention (*n* = 1614 staff from 66 workplaces)

	Number of strategies used at least sometimes		Strategy usage (0–4)	
	Unadjusted ^a^	Adjusted ^b^	Unadjusted ^a^	Adjusted ^b^
All strategies (*n* = 21)				
Pre-intervention Mean (SE)	9.56 (SE = 0.19)	9.56 (SE = 0.19)	1.48 (SE = 0.03)	1.48 (SE = 0.03)
During-intervention Mean (SE)	12.01 (SE = 0.21)	12.01 (SE = 0.21)	1.84 (SE = 0.03)	1.84 (SE = 0.03)
Change (95%CI)	2.45 (2.18, 2.73)	2.45 (2.18, 2.73)	0.37 (0.33, 0.41)	0.37 (0.33, 0.41)
p	< 0.001	< 0.001	< 0.001	< 0.001
ICC (95% CI)	0.124 (0.078, 0.193)	0.099 (0.056, 0.170)	0.133 (0.085, 0.204)	0.116 (0.068, 0.193)
Move more strategies (*n* = 13)				
Pre-intervention Mean (SE)	6.17 (SE = 0.14)	6.21 (SE = 0.13)	1.55 (SE = 0.03)	1.55 (SE = 0.03)
During-intervention Mean (SE)	7.25 (SE = 0.15)	7.29 (SE = 0.14)	1.83 (SE = 0.04)	1.85 (SE = 0.03)
Change (95%CI)	1.08 (0.89, 1.27)	1.09 (0.90, 1.28)	0.28 (0.23, 0.33)	0.28 (0.23, 0.33)
p	< 0.001	< 0.001	< 0.001	< 0.001
ICC (95% CI)	0.153 (0.101, 0.227)	0.126 (0.076, 0.204)	0.158 (0.104, 0.232)	0.135 (0.082, 0.215)

^a^ Linear mixed models, with random intercept for workplace cluster and staff member (nested within workplace cluster), and fixed effects for timepoint (pre-intervention/during-intervention)

^b^ Linear mixed models additionally include fixed effects for: age; full time employment (yes/no); job category skill level [1–5]; small-medium enterprise (yes/no); public sector (yes/no); regional or remote staff in team (yes/no); call centre staff in team (yes/no); COVID impact on organisation (none (pre-pandemic) / general / high impact); organisational readiness (context score); champion has occupational health and safety role (yes/no); champion age; champion-reported team interest in health [1–5]; and, socioeconomic position of workplace location (Index of Relative Socioeconomic Advantage and Disadvantage percentile)

### Aim 3: associations of strategy usage and changes in strategy usage with behaviours

Strategy usage (0–4) during the intervention was significantly and favourably associated with behaviours at post-intervention — lower work sitting (-1.69% [95%CI: -1.89, -1.49]), higher work moving (0.64% [0.54, 0.74]), and lower prolonged sitting accumulation (-1.85% [-2.12, -1.59]) — as well as changes in these behaviours (Table 4). Similarly, changes in strategy usage (-4–4) were also significantly associated favourably with changes in behaviours (Table 4).

**Table 4:** Associations of staff strategy usage with post-intervention behaviours (*n* = 66 workplaces) and changes in behaviours (*n* = 60 workplaces) ^a^

	*n*	Sitting, % of workday		Moving, % of workday		% of sitting in prolonged bouts	
		b (95% CI) / χ ^2^(df)	*p*	b (95% CI)	*p*	b (95% CI)	*p*
***Post-intervention behaviours***							
Strategy usage (0–4)	647	-1.69 (-1.89, -1.49)	< 0.001	0.64 (0.54, 0.74)	< 0.001	-1.85 (-2.12, -1.59)	< 0.001
Move-more strategy usage (0–4)		-1.25 (-1.48, -1.02)	< 0.001	0.61 (0.49, 0.73)	< 0.001	-1.39 (-1.70, -1.08)	< 0.001
Other strategy usage (0–4)		-2.83 (-3.20, -2.46)	< 0.001	0.71 (0.52, 0.91)	< 0.001	-3.07 (-3.56, -2.57)	< 0.001
*move-more interaction		51.43 (1)	< 0.001	0.76 (1)	0.383	32.24 (1)	< 0.001
*strategy (1/2/…21) interaction ^b^		129.18 (20)	< 0.001	71.08 (20)	< 0.001	105.63 (20)	< 0.001
***Changes in behaviours***							
Strategy usage (0–4)	332	-0.82 (-1.04, -0.61)	< 0.001	0.38 (0.27, 0.50)	< 0.001	-0.55 (-0.91, -0.18)	0.003
Move-more strategy usage (0–4)		-0.66 (-0.92, -0.41)	< 0.001	0.39 (0.25, 0.52)	< 0.001	-0.31 (-0.74, 0.11)	0.145
Other strategy usage (0–4)		-1.26 (-1.67, -0.85)	< 0.001	0.38 (0.17, 0.60)	< 0.001	-1.17 (-1.86, -0.49)	0.001
*move more interaction		-0.66 (-0.92, -0.41)	< 0.001	0.39 (0.25, 0.52)	< 0.001	-0.31 (-0.74, 0.11)	0.145
*strategy (1/2/…21) interaction ^b^		26.12 (20)	0.162	10.54 (20)	0.957	26.03 (20)	0.165
Strategy usage change (-4–4)	332	-0.91 (-1.17, -0.66)	< 0.001	0.37 (0.23, 0.50)	< 0.001	-0.87 (-1.29, -0.44)	< 0.001
Move-more strategy usage change (-4–4)		-1.28 (-1.73, -0.83)	< 0.001	0.28 (0.04, 0.51)	0.023	-1.16 (-1.92, -0.40)	0.003
Other strategy usage change (-4–4)		-0.76 (-1.07, -0.46)	< 0.001	0.41 (0.25, 0.58)	< 0.001	-0.75 (-1.26, -0.23)	0.004
*move-more interaction		5.43 (1)	0.020	0.59 (1)	0.444	2.11 (1)	0.146
*strategy (1/2/…21) interaction ^b^		32.15 (20)	0.042	21.48 (20)	0.369	20.72 (20)	0.414

^a^ adjusted for public sector (yes/no), small-medium enterprise (yes/no), age, sex, job classification (employee / middle management / upper management)

^b^ estimates of effects of usage / usage changes of each strategy based on these interaction models are presented in Supplemental Tables 3 and 4

While the above results reflected what was true on average in terms of associations, the interactions revealed some significant differences between move more strategies and other strategies. The ‘move more’ strategies (compared with other strategies) were significantly weaker in their associations of usage with post-intervention sitting and prolonged accumulation of sitting (p for interaction < 0.05), and usage changes with sitting changes (Table 4). Similar, but non-significant, differences were seen in terms of associations of changes in strategy with post-intervention prolonged accumulation of sitting and changes outcomes. Interestingly, there was no significant or large difference between move more and other strategies in their associations of usage and usage changes with moving and moving changes (p for interaction all > 0.05, Table 4).

**Fig. 1:**
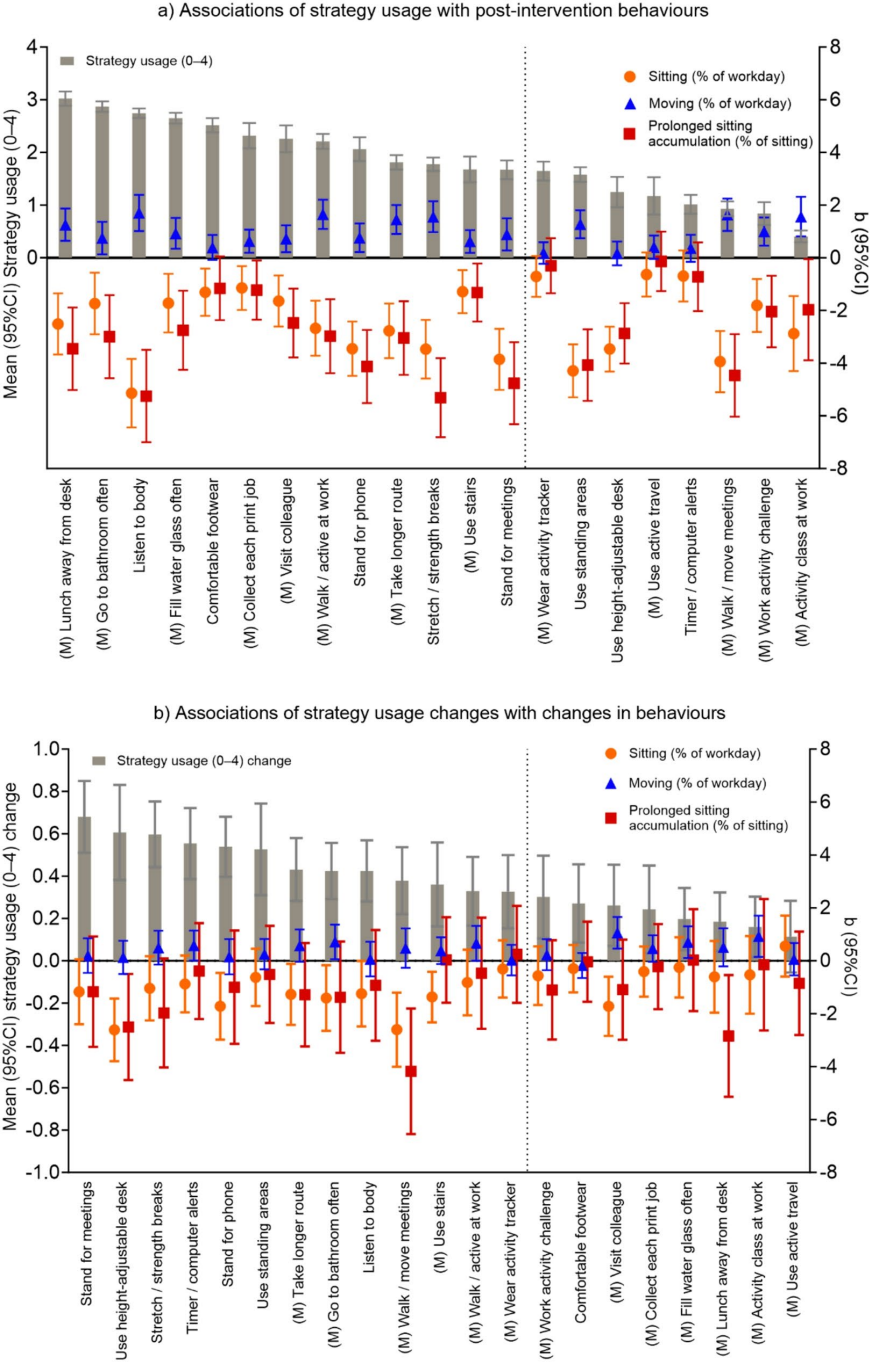
Associations of strategy usage with behaviours, ranked by mean strategy usage

The associations of strategy usage with post-intervention behaviours all varied significantly from one strategy to another (interactions all *p* < 0.001), as did the associations of changes in strategy usage with changes in sitting and prolonged sitting accumulation (Table 4), while other interactions did not reach statistical significance (*p* > 0.05). The associations conditional on strategy are presented numerically in Supplemental Tables 3 and 4 and are depicted graphically in Fig. 1. When significant interactions were detected, they always reflected different sizes of associations, rather than different directions of association. For every strategy, higher usage during the intervention tended to be associated with less sitting, more moving, and less prolonged accumulation of sitting (Supplemental Table 3). Strategy usage increases all tended towards more favourable sitting changes (Supplemental Table 4), but the conditional estimate seen for each strategy ranged from small and non-significant to larger and statistically significant (all with wide confidence intervals). Figure 1 depicts associations of individual strategy usage with each outcome, ranked by mean strategy usage, which helps to consider potential acceptability (high usage), modifiability (highly increased usage), and potential effectiveness (size of associations) simultaneously. One interesting intersection of these considerations are that the strategies that had high mean usage and/or strong increases in usage also showed strong associations of their usage (or increased usage) with all of the behaviours. This suggests they are acceptable, modifiable strategies that may be highly effective for improving sitting, moving, and prolonged accumulation of sitting. These included having lunch away from the desk, using the more distant bathroom, and listening to the body.

### Aim 4: combinations of strategies and behaviours

#### Sitting

**Fig. 2:**
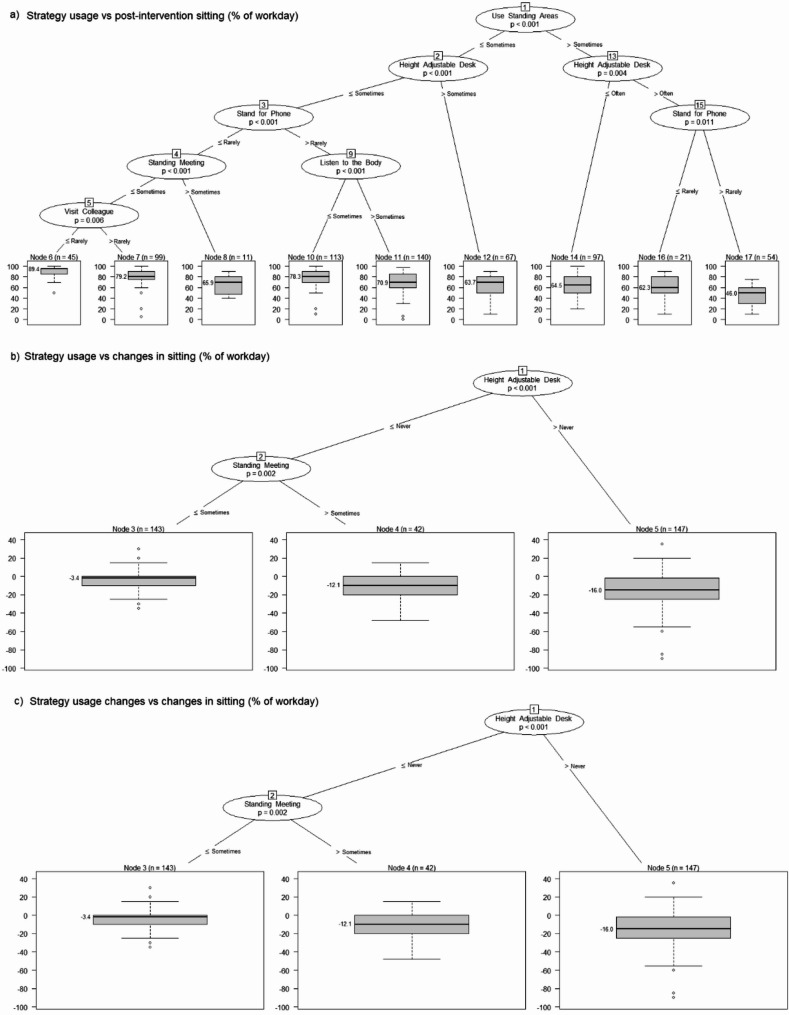
Conditional inference trees depicting prediction work sitting from staff strategy usage

Conditional inference trees (Fig. 2a) predicted post-intervention sitting based on the strategy usage during the intervention (Never / Rarely / Sometimes / Often / Very often or always) of six strategies. The first variable selected (top predictor) was usage of standing areas, followed by usage of height adjustable desks, then standing for phone calls. Workers who often (or more) used standing areas, very often (or more) used their height adjustable workstation, and sometimes (or more) stood for phone calls (*n* = 54, 8.3%) were the only group with mean sitting (46.0%) meeting the targeted ≤ 50%. Those who only sometimes (or less) used standing areas, height adjustable desks, and standing meetings, and rarely or never stood for phone calls or visited colleagues in person (*n* = 45, 7.0%) had the highest mean sitting (89.4%). The most populous combination (*n* = 140, 21.6%) used standing areas and height adjustable desks at most sometimes, but at least sometimes stood for calls, and often, very often or always listened to their body to shift posture. Those in this group achieved an intermediate mean sitting of 70.9%. Changes in sitting time (Fig. 2b) were predicted firstly by height adjustable desk usage and secondly by standing meetings. Those using height adjustable desks at all had the greatest reductions in sitting time (-16.0% of worktime) followed by those who never used this strategy but often to always used standing meetings (-12.1% of time), with the least mean sitting reductions (-3.4% of work time) seen in those remaining participants. Somewhat similarly, changes in height adjustable desk use were selected as the first predictor of sitting changes, followed by changes in working meetings, with benefits seen with increasing usage by two or more points (Fig. 2c).

#### Sitting accumulation

Post-intervention accumulation of sitting in long bouts was predicted first by stretching, then taking the longer route, visiting colleagues in person, standing for phone calls, and using standing areas (Supplemental Fig. 1a). Three groups had mean prolonged accumulation under 50%: a large group (*n* = 131, 20.2%) who stretched at least sometimes and used standing areas often to always (43.7% of sitting was prolonged); a very small group (*n* = 14, 2.2%) who rarely or never stretched but very often or always took the longer route; and, a small group (*n* = 26, 4.0%) who also never stretched and sometimes or less took the longer route, but did often to always visit colleagues in person and stand or move for calls. Changes in sitting accumulation were predicted by use of walking meetings (Supplemental Fig. 1b). Those who sometimes to always used walking meetings reduced prolonged accumulation by 20.6% of sitting time on average, with their counterparts reducing their prolonged sitting accumulation by a mean of 7.8%. Changes in walking meetings were the only predictor selected of changes in sitting accumulation, with the greatest improvements seen among those increasing their walking meeting usage at all (Supplemental Fig. 1c).

#### Moving

Post-intervention moving was predicted by usage of five strategies (Supplemental Fig. 2a). Firstly, taking the longer route places (top predictor), then stretching, walking meetings, visiting colleagues in person and finally standing for phone calls. Staff who very often or always took the longer route (*n* = 81, 12.5%) had a high mean moving (18.5% of worktime), just under that of the group with the highest mean moving (19.3%), who did not very often to always take the longer route but did stretch often or more, and had walking meetings at least sometimes (*n* = 44, 6.8%). The least moving (5% of the workday) was seen in a group (*n* = 56, 8.7%) with limited usage of all of the aforementioned strategies. Conditional inference trees did not select usage of any strategies during the intervention as predicting moving changes, but they did select usage changes in two strategies as predicting moving changes: visiting colleagues in person and computer alerts. The greatest moving increases (5.4% of workday) were seen in participants who increased, maintained or at least declined by no more than one point in their usage of visiting colleagues in person (top predictor), and increased their use of alerts by at least one point (Supplemental Fig. 2b).

## Discussion

The main evaluation of BeUpstanding previously demonstrated that the intervention significantly improves key work behaviours: sitting; prolonged sitting accumulation; and, moving [19]. In this present study, we described the sit less and move more strategies used by participants to make these changes. We found that the BeUpstanding intervention significantly increased: strategy usage, in terms of number of strategies used at least sometimes; mean strategy usage; and, usage of every strategy except active travel. We further linked strategy usage (and/or changes in strategy usage) with the behaviours seen after the intervention (and/or the behaviour changes that were made) and found some specific strategies whose usage showed particularly strong links with behaviour. Importantly these findings were drawn empirically from a nationwide intervention conducted in multiple diverse organisations across a broad range of industries and sectors, which is crucial as strategy usage is likely very sensitive to context. In doing so, this study adds greatly to the findings from previous research into sit less/move more strategies, all of which comes from single-organisation and/or single-sector interventions [15–17]. We also report on strategy usage prior to intervention, providing insights into what sit less/move more strategies are already acceptable (even before intervention) in Australian workplaces.

Findings suggest none of the 21 strategies measured should be excluded from any menu of suggested strategies as completely unacceptable: all were used at least sometimes during the intervention by > 10% of staff. Sixteen of the strategies were used at least sometimes by at least half of the participants, and the top five most common strategies were used at least sometimes by ≥ 75% of participants. These strategies — having lunch away from the desk, taking regular bathroom breaks, changing posture when feeling tired or uncomfortable (listening to the body), filling up the water bottle regularly, and using comfortable footwear — are suitable to emphasise in messaging based on their strong acceptability in addition to being minimal/no cost. Further supporting their wide appeal, some of these strategies were also heavily used by participants in other interventions, with having lunch away from the desk a common strategy reported in the Stand Up Lendlease study [17]. Interestingly, walking to see a colleague and stair use (which rely on being present in the workplace and building layout) both had very high usage (over 90%) in the Stand Up Lendlease trial [17] and were very common choices of Stand Up Victoria trial participants [16], while strategies had lower usage in the BeUpstanding trial (73% and 51% respectively). This illustrates the context-sensitive nature of strategies: it is likely not all participating workplaces had stairs, and walking to see a colleague was not necessarily possible given the timeline of the BeUpstanding implementation trial included the COVID-19 lockdowns and the associated increases in work-from-home arrangements [30]. Some tailoring of suggestions that are suitable for context may be useful where strategies require being in the workplace or given specific building features (such as access to stairs).

In addition to strategies that are highly acceptable based on their high usage, messaging could emphasise strategies where usage increased strongly over the course of the intervention, as these may be particularly modifiable. Height-adjustable workstation usage, for example, increased strongly, and accordingly appears modifiable and acceptable in contexts where they are or can be made available. None of the strategies merit exclusion from a potential menu of strategies as non-modifiable, as usage of every strategy increased to some extent. The single exception was possibly active travel, which showed the least increase and is subject to numerous environmental and other constraints. While active travel did not significantly increase in BeUpstanding, there was no requirement for workplaces to include this as a particular intervention focus. The lack of change in BeUpstanding should not be taken as evidence active travel cannot be increased through other interventions, or negates the need to continue promoting active travel in a more general health promotion context, including by providing the relevant support infrastructure such as showers and safe storage.

Strategy usage, and changes in strategy usage, were significantly associated with more favourable levels and changes in sitting at work, moving at work, and the accumulation of prolonged sitting at work. However, sometimes different strategies varied in the extent to which their usage (or usage increases) were associated with these behaviours and/or behaviour changes. Accordingly, conditional inference mostly were able to select one or more strategies as predicting outcomes amounts and changes. Importantly, findings did not provide any firm recommendations to avoid any of the strategies. None of the strategies were counterproductive; rather, the strength of their associations sometimes differed, but not always in the same way for every outcome. Findings do, however, provide some basis for emphasising some strategies over others when presenting a smaller set of choices: something that both researchers (in designing interventions) and workplaces (in promoting strategies) may consider. In addition to considering appeal, and modifiability, relevant considerations should include emphasising strategies strongly linked with behaviours, to help ensure effectiveness.

Some of the strategies that may merit emphasis on a menu of strategies as potentially highly effective (based on their emergence in decision trees or the interaction findings) require environmental supports (using standing areas; using height adjustable desks; standing for phone calls). Others did not, but likely require a supportive culture for movement, such as listening to the body, stretching or taking strength breaks, walking meetings, lunch away from the desk, taking the longer route places, and visiting colleagues in person. Height adjustable workstations were the top predictor of reducing sitting: a finding consistent with systematic review evidence showing height-adjustable workstations to be a highly useful component to include when targeting occupational sitting [12–14], especially alongside approaches used in BeUpstanding such as raising awareness and building a supportive culture for change. Where able to be made available, this strategy is strongly recommended based on both effectiveness and strong uptake, noting further evidence on the cost-effectiveness of sit-stand workstations is still needed [31]. Recommendations for strategies to improve moving in workplace sit less/move more interventions were less clear. Moving has known benefits [19], even at a light intensity [6], but is often only increased to a small degree by sit less/move more interventions [11, 32]. This includes for BeUpstanding where the national implementation trial showed self-report moving increased by an average of 1.3% of worktime (≈ 6 min per 8 h working) [19]. Here, strategies that emerged as particularly important for moving (albeit not for moving changes) were taking the longer route places, visiting colleagues in person, stretching, walking meetings, standing for phone calls, and using timer / computer alerts. These potentially highly effective moving strategies may require strong cultural support for regularly leaving the desk to move. While their comparative benefit relative to other strategies is uncertain and requires further research, due to the high importance (and historic low success) of increasing moving, it is recommended that regular usage of these moving strategies be promoted, coupled with strong cultural support to leave the desk.

The strategies with limited evidence of effectiveness in terms of the work behaviours targeted in BeUpstanding were active travel and wearing an activity tracker. Active travel has multilevel benefits to the individual, economy, and environment [33], but not unexpectedly showed limited associations with sitting less and moving more *during* work time, and as such is lower priority to emphasise to help people sit less and move more at work. How often workers wore an activity tracker to track their physical activity or sitting (not necessarily during work) had a comparatively limited relationship with the work behaviours relative to the other strategies. Activity trackers do increase physical activity in other intervention contexts [34], may support reductions in prolonged sedentary time through several features such as movement prompts and daily goals [35], and are a growing part of everyday life for people, representing a market that is expected to continue to expand to an estimated US$56.82bn by 2029 [36]. Rather than avoiding activity trackers as a strategy, the relevant issue may be determining how sit less/move more interventions can better leverage the behaviour change potential of these devices in the context of a multilevel approach. To better understand why usage of some strategies may be more effective than others, future studies could use qualitative approaches to enquire about staff perspectives.

A key strength of this study was that staff from multiple organisations and from a diversity of locations, sizes, and sectors were included, enhancing the generalisability of the findings. The key limitation was the self-report of both strategy usage and behaviour. Further, the measure of strategy usage is relatively crude and unlikely to pick up nuances in the actual strategy usage (e.g., asking about stair use does not measure how many flights of stairs). Data were only used from the list of strategies asked of participants. Although this list had been iteratively developed from previous studies [16, 17, 23, 26], it is likely not all strategies used by participants were captured. Further, although there was adjustment for clustering and potential confounders, there may be other unmeasured factors influencing associations. It will also be important to explore if there are variations in strategy usage across different workplaces and job roles as well as by work-from-home status. It should be noted that the conditional inference trees used do not correct for the clustering of staff within teams or workplaces. This, and other methodological differences, partly explain why different results can be obtained based on the interaction analysis compared with the conditional inference trees.

## Conclusion

This study provided novel evidence from a wide diversity of organisations on strategies desk-based workers use to sit less and move more, the impact of the BeUpstanding intervention on strategy usage, and the relationships of strategy usage with work behaviours. Findings suggest that while some strategies could be emphasised, none of the strategies measured should be excluded from a menu of strategies to support desk workers to sit less and move more, thus allowing workers to choose what best suits them and their context.

## Electronic supplementary material

Below is the link to the electronic supplementary material.

Supplementary Material 1

## Data Availability

The datasets generated and analysed during the current study are available from the corresponding author on reasonable request.
