# Supplementary material for: Usage and effectiveness of strategies to sit less and move more: evaluation of the BeUpstanding™ national implementation trial
Source: Int J Behav Nutr Phys Act. 2025 May 28;22:63. doi: 10.1186/s12966-025-01761-4 (PMC12117936; doi:10.1186/s12966-025-01761-4)
Supplement: Supplementary file 1 — Supplementary Material 1 [file 12966_2025_1761_MOESM1_ESM.docx]

**Supplemental Material**

**Supplemental Table 1**: Strategy questionnaire items collected in pre-and post- intervention surveys in BeUpstanding, with their scale reliability (Cronbach's alpha) for strategy usage **^a^**

| **Questionnaire item / Scale** | **Shortened name** | **Cronbach’s alpha ^b^** | |
| --- | --- | --- | --- |
|  |  | **Pre intervention (n=1701)** | **Post intervention (n=681)** |
| Test scale: Strategy Usage (21 items) |  | 0.7624 | 0.7898 |
| Test scale: Move-more strategy Usage (13 items) |  | 0.6835 | 0.6885 |
| Test scale: Remaining strategy Usage (8 items) |  | 0.6399 | 0.676 |
| ***Move-more strategy items*** |  |  |  |
| 1. Used bathroom breaks as an opportunity to sit less and move more | (M) Go to bathroom often | 0.7464 | 0.7794 |
| 1. Picked up the printing after each job rather than letting the jobs pile up | (M) Collect each print job | 0.7538 | 0.784 |
| 1. Done active travel to work (e.g., walked, cycled, used public transport) | (M) Use active travel | 0.7597 | 0.7951 |
| 1. Used an activity monitor / wearable device to track my activity and/or sitting time | (M) Wear activity tracker | 0.7628 | 0.7923 |
| 1. Used the stairs instead of taking the lift | (M) Use stairs | 0.7553 | 0.7888 |
| 1. Participated in an activity class (e.g., yoga, Pilates) at my workplace | (M) Activity class at work | 0.7579 | 0.7856 |
| 1. Had a walking meeting / did a "walk and talk" | (M) Walk / move meetings | 0.7531 | 0.7765 |
| 1. Went for a walk / did some physical activity during the work day (e.g., during my lunch break) | (M) Walk / active at work | 0.7467 | 0.7744 |
| 1. Taken the longer route (e.g., walked to the printer, bathroom, or bin etc. that was further away from my desk) | (M) Take longer route | 0.7411 | 0.7705 |
| 1. Participated in an activity challenge with my workplace (e.g., Global Corporate Challenge, Fun Run) | (M) Work activity challenge | 0.7589 | 0.7819 |
| 1. Walked to talk to a colleague rather than sending them an email or phoning | (M) Visit colleague | 0.7512 | 0.7786 |
| 1. Used filling up my water bottle or glass as a strategy to get up regularly | (M) Fill water glass often | 0.7506 | 0.7802 |
| 1. Taken my breaks (e.g., lunch) away from my desk / workstation | (M) Lunch away from desk | 0.7602 | 0.7862 |
| ***Other strategy items*** |  |  |  |
| 1. Worn comfortable shoes to work to be able to stand and move more easily | Comfortable footwear | 0.7526 | 0.7831 |
| 1. Used a height adjustable desk to alternate between sitting and standing | Use height adjustable desk | 0.7562 | 0.7817 |
| 1. Changed posture either by sitting, standing or moving when I was feeling tired or uncomfortable | Listen to body | 0.7461 | 0.776 |
| 1. Utilised standing areas in my workplace | Use standing areas | 0.7477 | 0.7727 |
| 1. Taken a break to do stretches or use my muscles (e.g., squats) | Stretch / strength breaks | 0.7488 | 0.7765 |
| 1. Used a time / alarm / computer or device based prompt to remind me to stand up and move more regularly | Timer / computer alerts | 0.7589 | 0.7865 |
| 1. Stood up and (if possible) moved around when taking a phone call | Stand for phone | 0.7574 | 0.7841 |
| 1. Stood up during a meeting | Stand for meetings | 0.7562 | 0.778 |

^a^ Full question asked before each item is as follows: “These questions ask about how often you used various strategies to stand up, sit less and move more at work over the **last month**. If you have not used these strategies please indicate ‘never’ in the questions below. If they are not applicable, please select the < not applicable > option. In the past month I have... *(Please choose the most appropriate option)”. Response options given are: Never, Rarely, Sometimes, Often, Very often/always, < not applicable >*

^b^ Cronbach’s alpha for scale reliability of the test scales / Cronbach’s alpha for strategy usage scale if item is removed

**Supplemental Table 2**: Odds of teams and staff being included in the analysis (yes/no) based on workplace and staff characteristics

| **Characteristic** | **n** | **Odds Ratio (95% CI)** | **p** |
| --- | --- | --- | --- |
| **Teams** |  |  |  |
| State (collapsed) | 94 |  | *0.682* |
| NSW/ACT |  | 1 (ref) |  |
| VIC |  | 1.08 (0.21, 5.45) | 0.927 |
| QLD |  | 0.68 (0.21, 2.23) | 0.524 |
| SA |  | 0.39 (0.06, 2.37) | 0.308 |
| WA/TAS/NT |  | 2.06 (0.20, 20.96) | 0.540 |
| Small-medium enterprise, yes vs no | 94 | 12.00 (1.53, 94.34) | 0.018 |
| Sector | 94 |  | *0.053* |
| Government / public |  | 1.00 (ref) |  |
| Nonprofit |  | 7.80 (0.94, 64.74) | 0.057 |
| Private |  | 2.60 (0.90, 7.53) | 0.078 |
| Regional staff included, yes vs no | 94 | 0.44 (0.17, 1.13) | 0.089 |
| Call-centre staff included, yes vs no | 94 | 0.06 (0.01, 0.53) | 0.012 |
| **Staff** |  |  |  |
| Age, per 10 years | 2043 | 1.27 (1.02, 1.57) | 0.030 |
| Female, yes vs no | 2085 | 0.98 (0.71, 1.38) | 0.940 |
| Job classification | 2043 |  |  |
| Employee |  | 1 (ref) |  |
| Middle management |  | 1.74 (0.80, 3.79) |  |
| Upper management |  | 1.87 (1.11, 3.14) |  |
| Full-time employment | 2043 |  |  |
| No |  | 1 (ref) |  |
| Yes |  | 1.22 (0.72, 2.06) | 0.467 |
| Work hours, per 10 hours/week |  | 1.07 (0.85, 1.35) | 0.576 |

Table presents Odds ratio, 95% CI and p from mixed effects logistic regression models, with outcome variables of included in the analysis (yes/no) for both the team-level analysis (n=94) and staff-level analysis (n=2043–2085).

**Supplemental Table 3**: Associations of strategy usage during the program (0-4) with post-program behaviours and changes in behaviours ^a^

|  | Post-intervention behaviours (n=66 workplaces, 647 staff) | | | Behaviour changes (n=60 workplaces, 332 staff) | | |
| --- | --- | --- | --- | --- | --- | --- |
|  | Sitting, % of workday | Moving,% of workday | % of Sitting Prolonged | Sitting,  % of workday | Moving,  % of workday | % of Sitting Prolonged |
| ***Move more strategy*** |  |  |  |  |  |  |
| Go to bathroom often | **-1.73 (-2.90, -0.57)** | **0.75 (0.13, 1.37)** | **-3.00 (-4.57, -1.42)** | **-2.14 (-3.42, -0.86)** | **0.91 (0.23, 1.58)** | 0.01 (-2.14, 2.16) |
| Collect each print job | **-1.15 (-1.98, -0.31)** | **0.63 (0.19, 1.07)** | **-1.23 (-2.35, -0.11)** | -0.50 (-1.42, 0.42) | 0.47 (-0.02, 0.96) | -0.14 (-1.69, 1.41) |
| Use active travel | **-0.63 (-1.47, 0.21)** | 0.40 (-0.04, 0.85) | -0.13 (-1.26, 0.99) | -0.48 (-1.38, 0.43) | **0.66 (0.18, 1.13)** | -0.11 (-1.63, 1.41) |
| Activity Tracker | -0.71 (-1.48, 0.07) | 0.18 (-0.23, 0.59) | -0.30 (-1.34, 0.75) | -0.69 (-1.53, 0.16) | 0.28 (-0.17, 0.73) | -0.13 (-1.56, 1.29) |
| Use stairs | **-1.29 (-2.11, -0.47)** | **0.62 (0.19, 1.06)** | **-1.32 (-2.42, -0.21)** | -0.61 (-1.52, 0.29) | 0.32 (-0.16, 0.80) | -0.04 (-1.56, 1.48) |
| Activity class at work | **-2.88 (-4.30, -1.45)** | **1.56 (0.81, 2.32)** | **-1.97 (-3.89, -0.05)** | -0.28 (-1.84, 1.27) | **1.02 (0.20, 1.84)** | 0.17 (-2.45, 2.79) |
| Walk / move meetings | **-3.94 (-5.10, -2.78)** | **1.64 (1.02, 2.25)** | **-4.47 (-6.03, -2.90)** | **-2.58 (-3.93, -1.23)** | 0.44 (-0.28, 1.16) | **-3.72 (-5.99, -1.44)** |
| Walk / active at work | **-2.67 (-3.72, -1.63)** | **1.65 (1.10, 2.20)** | **-2.97 (-4.38, -1.57)** | -0.72 (-1.85, 0.41) | **0.61 (0.02, 1.21)** | 0.49 (-1.41, 2.39) |
| Take longer route | **-2.77 (-3.81, -1.74)** | **1.45 (0.91, 2.00)** | **-3.04 (-4.44, -1.65)** | **-1.45 (-2.56, -0.34)** | **0.74 (0.15, 1.32)** | 0.06 (-1.80, 1.93) |
| Work activity challenge | **-1.81 (-2.81, -0.80)** | **1.00 (0.47, 1.53)** | **-2.04 (-3.40, -0.68)** | -0.75 (-1.83, 0.33) | 0.28 (-0.29, 0.85) | **-2.01 (-3.82, -0.20)** |
| Visit colleague | **-1.64 (-2.61, -0.67)** | **0.73 (0.21, 1.24)** | **-2.47 (-3.78, -1.17)** | **-1.29 (-2.33, -0.24)** | **0.63 (0.07, 1.18)** | -0.74 (-2.50, 1.02) |
| Fill water glass often | **-1.72 (-2.83, -0.61)** | **0.93 (0.34, 1.52)** | **-2.75 (-4.25, -1.25)** | -0.41 (-1.62, 0.80) | 0.56 (-0.08, 1.20) | -0.16 (-2.20, 1.87) |
| Lunch away from desk | **-2.51 (-3.67, -1.35)** | **1.26 (0.64, 1.87)** | **-3.45 (-5.02, -1.89)** | **-1.27 (-2.54, -0.01)** | **0.70 (0.03, 1.37)** | -1.44 (-3.57, 0.69) |
| ***Stand up / sit less strategy*** |  |  |  |  |  |  |
| Comfortable footwear | **-3.46 (-4.31, -2.61)** | 0.17 (-0.28, 0.62) | **-2.87 (-4.02, -1.72)** | **-1.78 (-2.70, -0.86)** | 0.23 (-0.26, 0.71) | **-1.95 (-3.50, -0.40)** |
| Use height-adjustable desk | **-1.31 (-2.21, -0.41)** | 0.39 (-0.08, 0.87) | -1.16 (-2.37, 0.05) | -0.52 (-1.51, 0.47) | 0.22 (-0.30, 0.74) | 0.35 (-1.31, 2.02) |
| Listen to body | **-5.14 (-6.44, -3.84)** | **1.70 (1.02, 2.39)** | **-5.25 (-7.00, -3.50)** | **-2.48 (-3.94, -1.02)** | **0.80 (0.03, 1.57)** | -1.24 (-3.69, 1.21) |
| Use standing areas | **-4.29 (-5.30, -3.28)** | **1.28 (0.75, 1.81)** | **-4.07 (-5.43, -2.72)** | **-1.57 (-2.70, -0.44)** | **0.63 (0.03, 1.23)** | -0.21 (-2.11, 1.69) |
| Stretch / strength breaks | **-3.47 (-4.58, -2.36)** | **1.56 (0.97, 2.15)** | **-5.31 (-6.81, -3.81)** | -0.78 (-2.07, 0.51) | 0.43 (-0.25, 1.11) | -1.19 (-3.36, 0.98) |
| Timer / computer alerts | -0.69 (-1.66, 0.28) | 0.36 (-0.15, 0.87) | -0.72 (-2.03, 0.58) | -0.68 (-1.76, 0.40) | 0.55 (-0.02, 1.13) | -0.04 (-1.86, 1.79) |
| Stand for phone | **-3.45 (-4.48, -2.42)** | **0.76 (0.22, 1.31)** | **-4.13 (-5.51, -2.74)** | **-1.69 (-2.83, -0.56)** | 0.45 (-0.15, 1.05) | **-2.41 (-4.32, -0.50)** |
| Stand for meetings | **-3.85 (-5.01, -2.70)** | **0.89 (0.28, 1.50)** | **-4.76 (-6.32, -3.20)** | **-1.53 (-2.82, -0.25)** | 0.24 (-0.44, 0.92) | **-2.59 (-4.75, -0.42)** |

Bold denotes p<0.05

^a^ Model includes random intercept for workplace cluster and strategy (which is repeated for individual staff members), as well as fixed effects for strategy change (-4 to 4) and strategy (1-19) along with their interaction, public sector (yes/no), small-medium enterprise (yes/no), age, sex, and job classification (employee / middle management / upper management

**Supplemental Table 4:** Associations of strategy usage changes with changes in behaviours ^a^

|  | Changes (n=60 workplaces, 325 staff) | | |
| --- | --- | --- | --- |
|  | Sitting, % of workday | Moving,% of workday | % of Sitting Prolonged |
| ***Move more strategy*** |  |  |  |
| Go to bathroom often | **-1.41 (-2.65, -0.17)** | **0.71 (0.05, 1.37)** | -1.38 (-3.48, 0.72) |
| Collect each print job | -0.41 (-1.36, 0.54) | 0.46 (-0.04, 0.97) | -0.22 (-1.83, 1.39) |
| Use active travel | 0.55 (-0.60, 1.71) | 0.06 (-0.55, 0.67) | -0.85 (-2.80, 1.10) |
| Activity Tracker | -0.31 (-1.39, 0.77) | 0.03 (-0.55, 0.60) | 0.24 (-1.59, 2.08) |
| Use stairs | -1.37 (-2.32, -0.41) | 0.38 (-0.12, 0.89) | 0.03 (-1.59, 1.65) |
| Activity class at work | -0.53 (-2.00, 0.94) | **0.93 (0.15, 1.70)** | -0.15 (-2.63, 2.34) |
| Walk / move meetings | **-2.60 (-4.00, -1.20)** | 0.48 (-0.27, 1.22) | **-4.18 (-6.55, -1.80)** |
| Walk / active at work | -0.82 (-2.06, 0.42) | 0.66 (-0.00, 1.31) | -0.47 (-2.57, 1.63) |
| Take longer route | **-1.27 (-2.42, -0.12)** | 0.57 (-0.04, 1.18) | -1.28 (-3.24, 0.67) |
| Work activity challenge | -0.56 (-1.67, 0.54) | 0.23 (-0.36, 0.82) | -1.10 (-2.98, 0.78) |
| Visit colleague | **-1.72 (-2.84, -0.60)** | **1.06 (0.47, 1.65)** | -1.09 (-2.99, 0.80) |
| Fill water glass often | -0.25 (-1.39, 0.89) | **0.70 (0.10, 1.31)** | 0.03 (-1.90, 1.95) |
| Lunch away from desk | -0.61 (-1.96, 0.75) | 0.51 (-0.21, 1.23) | **-2.84 (-5.14, -0.54)** |
| ***Stand up / sit less strategy*** |  |  |  |
| Comfortable footwear | **-2.61 (-3.79, -1.43)** | 0.13 (-0.50, 0.75) | **-2.50 (-4.51, -0.50)** |
| Use height-adjustable desk | -0.30 (-1.19, 0.60) | -0.18 (-0.65, 0.29) | -0.04 (-1.55, 1.48) |
| Listen to body | **-1.24 (-2.48, -0.01)** | 0.06 (-0.59, 0.72) | -0.93 (-3.02, 1.16) |
| Use standing areas | -0.63 (-1.71, 0.46) | 0.25 (-0.32, 0.83) | -0.51 (-2.35, 1.32) |
| Stretch / strength breaks | -1.04 (-2.26, 0.17) | 0.49 (-0.15, 1.14) | -1.97 (-4.03, 0.08) |
| Timer / computer alerts | -0.88 (-1.95, 0.19) | **0.58 (0.01, 1.14)** | -0.39 (-2.21, 1.42) |
| Stand for phone | **-1.72 (-2.98, -0.46)** | 0.15 (-0.52, 0.82) | -1.00 (-3.14, 1.14) |
| Stand for meetings | -1.17 (-2.40, 0.06) | 0.19 (-0.46, 0.85) | -1.17 (-3.26, 0.92) |

Model includes random intercept for workplace cluster and strategy (which is repeated for individual staff members), as well as fixed effects for strategy change (-4 to 4) and strategy (1-19) along with their interaction, public sector (yes/no), small-medium enterprise (yes/no), age, sex, and job classification (employee / middle management / upper management)

**Supplemental Figure 1:** Conditional inference trees depicting prediction of prolonged sitting accumulation from staff strategy usage


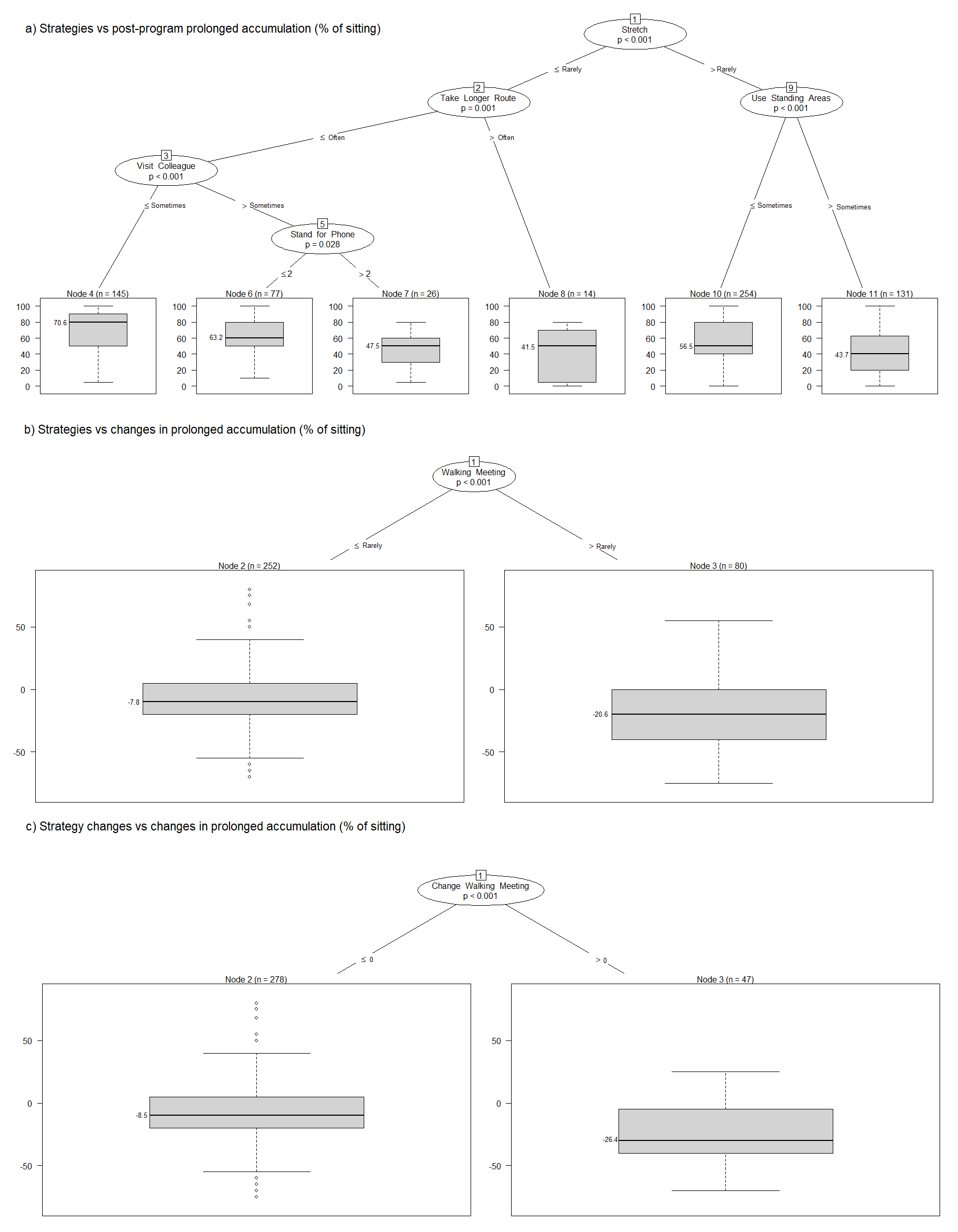


**Supplemental Figure 2:** Conditional inference trees depicting prediction of work moving from staff strategy usage ^a^


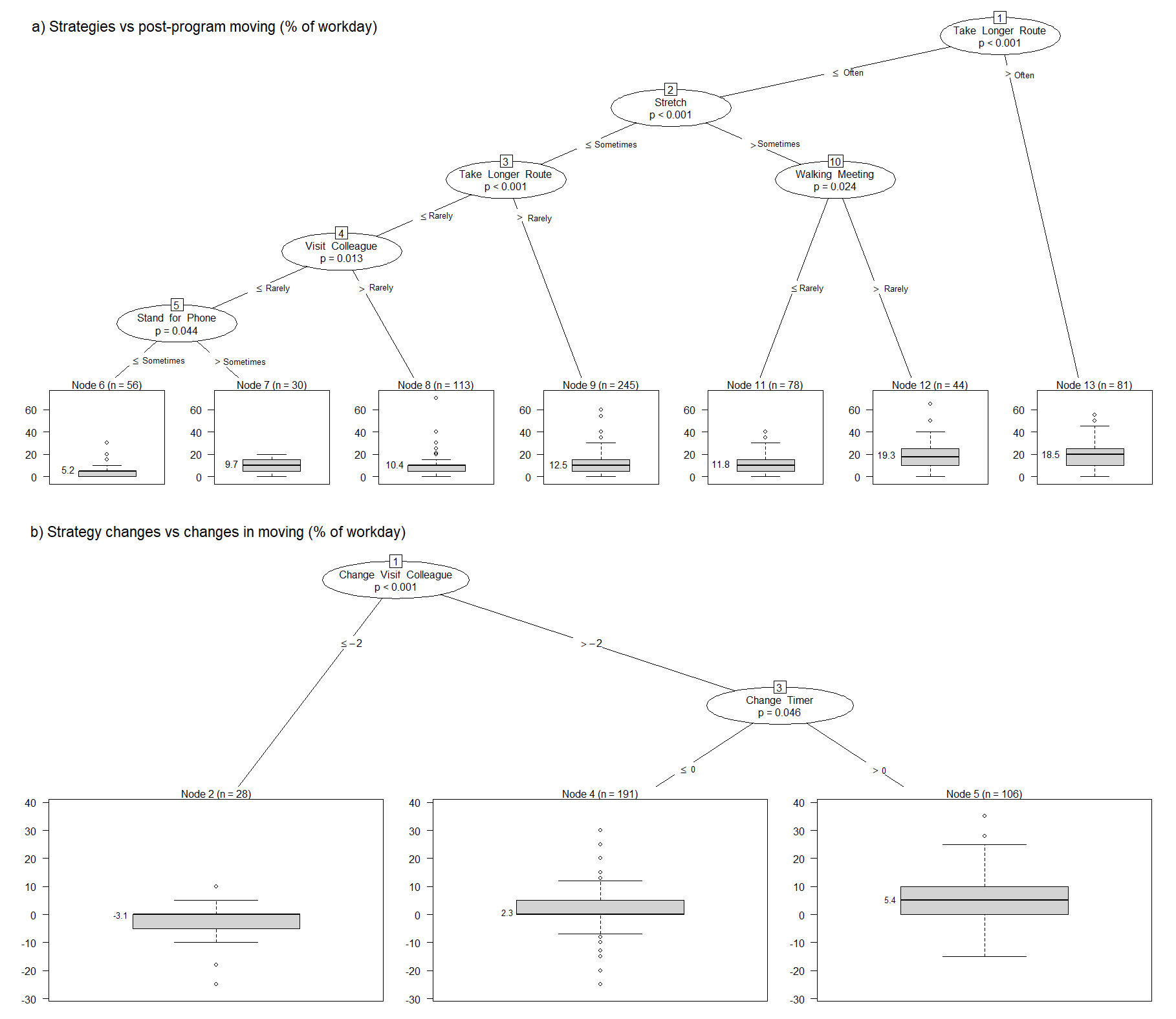


^a^ Tree not displayed for the prediction of changes in moving by strategy use during the program where no predictors were found and trees stopped at Node 1.
